# Chikungunya Virus nsP2 Impairs MDA5/RIG-I-Mediated Induction of NF-κB Promoter Activation: A Potential Target for Virus-Specific Therapeutics

**DOI:** 10.4014/jmb.2012.12005

**Published:** 2020-12-16

**Authors:** Sojung Bae, Jeong Yoon Lee,, Jinjong Myoung

**Affiliations:** Korea Zoonosis Research Institute, Department of Bioactive Material Science and Genetic Engineering Research Institute, Jeonbuk National University, Jeonju 54531, Republic of Korea

**Keywords:** Chikungunya virus, nsP2, type I interferon, evasion

## Abstract

Chikungunya virus (CHIKV) was first identified in 1952 as a causative agent of outbreaks. CHIKV is transmitted by two mosquito species, *Aedes aegypti* and *A. albopictus*. Symptoms after CHIKV infection in human are typically fever and joint pain, but can also include headache, muscle pain, joint swelling, polyarthralgia, and rash. CHIKV is an enveloped single-stranded, positive-sense RNA virus with a diameter of approximately 70 nm. The pathogenesis of CHIKV infection and the mechanism by which the virus evades the innate immune system remain poorly understood. Moreover, little is known about the roles of CHIKV-encoded genes in the viral evasion of host immune responses, especially type I interferon (IFN) responses. Therefore, in the present study, we screened CHIKV-encoded genes for their regulatory effect on the activation of nuclear factor kappa B (NF-κB), a critical transcription factor for the optimal activation of IFN-β. Among others, nonstructural protein 2 (nsP2) strongly inhibited melanoma differentiation-associated protein 5 (MDA5)-mediated induction of the NF-κB pathway in a dose-dependent manner. Elucidation of the detailed mechanisms of nsP2-mediated inhibition of the MDA5/RIG-I signaling pathway is anticipated to contribute to the development of virus-specific therapeutics against CHIKV infection.

## Introduction

Chikungunya virus (CHIKV) is an enveloped virus with a single-stranded RNA genome with positive polarity. CHIKV belongs to the genus *Alphavirus* of the *Togaviridae* family [1]. The name Chikungunya is derived from a word in the Kimakonde language meaning “that which bends up.” CHIKV is transmitted by mosquitoes and causes sporadic outbreaks worldwide, the first of which occurred in Africa in 1952, followed by epidemic outbreaks in Africa, Asia, and America [2]. The most common vectors for CHIKV transmission are two mosquito species, *Aedes aegypti* and *A. albopictus*. Symptoms include fever, headache, muscle pain, joint pain, and arthralgia [3]. The CHIKV genome is approximately 12 kb in size, with a diameter of around 70 nm. It encodes four nonstructural proteins (nsP1–4) for viral replication and six structural proteins (C, E3, E2, 6K/TF, and E1) in the subgenomic RNA [4]. E1 and E2 heterodimers bind to the cellular receptor on the target cells, mediating membrane fusion and viral entry. 6K and E3 function to translocate the precursor envelope proteins (E1 and E2) into the endoplasmic reticulum. Transframe (TF) protein is expressed via a frameshift event in the gene ofnd both TF and 6K contribute to viral budding.

The innate immune system senses pathogens by recognizing pathogen-associated molecular patterns (PAMPs) such as melanoma differentiation-associated protein 5 (MDA5) and retinoic acid-inducible gene-I (RIG-I), which detect double-stranded (ds) RNAs in infected cells [5-7]. MDA5 and RIG-I, upon ligand binding, undergo conformational changes, mediating the activation of downstream signaling through CARD-CARD interactions [8-11]. Mitochondrial antiviral-signaling (MAVS) contains an N-terminal CARD that interacts with the CARDs of MDA5 and RIG-I, resulting in the activation of MAVS, which subsequently activates the downstream kinase proteins TBK1 and IKKε. In turn, these proteins activate interferon regulator factor (IRF)-3 and nuclear factor kappa B (NF-κB) [12-14].

Activated IRF3 or IRF7 and NF-κB together induce type I interferon (IFN)-β production; secreted IFN-β binds to IFN receptors and directly activates the Janus kinase/signal transducers and activators of transcription (JAK-STAT) signaling pathway to express IFN-stimulated genes (ISGs) [15, 16]. However, the pathogenesis of CHIKV infection and the mechanism by which the virus evades the innate immune system remain poorly understood. Moreover, little is known about the roles of CHIKV-encoded genes in the evasion of host immune responses, especially type I IFN responses. Therefore, in this study, we screened CHIKV-encoded genes for their regulatory effect on the activation of NF-κB, a critical transcription factor for the optimal activation of IFN-β. It is interesting to note that CHIKV-encoded nsP2, E1, and E2 proteins strongly downregulate almost all signaling molecules involved in the MDA5/RIG-I pathway, namely, MDA5, RIG-I, MAVS, IKKε, and TBK1. Subsequent studies show that nsP2 inhibits MDA5-induced activation of the NF-κB promoter activities in a dose-responsive manner while its inhibitory activity is retained regardless of the presence/absence of tag (3X FLAG) and its position (either N-term or C-term), suggesting that nsP2 is a strong antagonist of IFN-β induction upon viral infection in the cells. Further studies are warranted to fathom the underlying mechanisms of nsP2-mediated inhibition of IFN-β expression and will contribute to the development of vaccines and therapeutics against CHIKV.

## Materials and Methods

### Cells

HEK293T cells were acquired from the American Type Culture Collection (USA). Cells were maintained at 37°C in a humidifying 5% CO_2_ incubator with Dulbecco’s modified Eagle’s medium (Welgene, Republic of Korea) supplemented with 10% fetal bovine serum (Welgene) and 1% penicillin/streptomycin (Thermo Fisher Scientific, USA).

### Reagents

Opti-MEM and the Pierce BCA Assay Kit were purchased from Thermo Fisher Scientific. Polyethylenimine (PEI) and complete Mini Protease Inhibitor Cocktail were purchased from Millipore Sigma (USA). The Luciferase Assay and Beta-Glo Assay systems were obtained from Promega (USA). EZ-Cytox was obtained from DoGenBio (Republic of Korea). The 4× Laemmli sample buffer and 2-mercatoethanol were obtained from Bio-Rad (USA). Amersham ECL western blotting detection reagent, Amersham ECL Prime western blotting detection reagent, and Amersham Protran 0.45 NC (nitrocellulose) western blotting membranes were purchased from GE Healthcare Life Sciences (USA) [17, 18]. Mouse monoclonal anti-FLAG was purchased from Sigma-Aldrich (USA). Mouse monoclonal anti- hemagglutinin (HA), rabbit monoclonal anti-HA, and rabbit anti-GAPDH antibodies conjugated with horseradish peroxidase (HRP) were purchased from Cell Signaling Technology (USA). Anti-mouse IgG conjugated with HRP and anti-rabbit IgG conjugated with HRP were obtained from Santa Cruz Biotechnology (USA). Monoclonal anti-CHIKV-nsP2 and anti-MAVS antibody were purchased from Abgenex (India) and Cell Signaling (USA), respectively. Pfu Plus DNA polymerase was obtained from Elpis Biotech (Republic of Korea). The restriction enzymes SbfI and ApaI were purchased from Enzynomics (Republic of Korea). T4 DNA ligase was obtained from New England Biolabs (USA).

### Plasmid Construction

The multiple cloning site (MCS) of the pcDNA3.1-Hygro (+) vector was modified by introducing the linkers for 3X FLAG and 3X GGGGS using NheI and Pmel, respectively. The modified vector was named pcDNA3.1-Hygro-3X FLAG-GS3 and had the following sequence in the multi-cloning site: 5’-GCTAGCGCCACCATGGACTACAAGGACCACGACGGTGACTACAAGGACCACGACATCGACTACAAGGACGACGACGACAAGCTTTCTGGTGGCGGTGGCTCGGGCGGAGGTGGGTCGGGTGGCGGCGGATCCTGCAGGCGCGCCGAATTCGAAAGCGCTATCGATATCGATGGCGCCTGGCCAGACCATCAGTCGAGTGGCGCCACTGGACTAATGGTCCGTACGCTCGACTGTACAGGCCGGCCTCAGGTTAACACCGGTACCTCAGCCCGGGCGGCCGCATG CGGGCCCCTCGAGTCTAGAGTTTAAAC-3’. Complementary DNA (cDNA) was prepared using M-MLV reverse transcriptase (Promega) from CHIKV genomic RNA, according to the manufacturer’s instructions. CHIKV genes were amplified from the cDNA by polymerase chain reaction (PCR) using Pfu polymerase (Elpis Biotech, Republic of Korea). Each amplified gene was cloned into pcDNA3.1-Hygro-JY4-GS3, pcDNA3.1-Hygro-JY4-3X FLAG-GS3, and pcDNA3.1-Hygro-JY4-3X FLAG-GS3 vectors using the SbfI and ApaI restriction enzymes (Enzynomics). The sequences of the primers used in this study are provided in Table 1.

### Transfection and Luciferase Reporter Assays

HEK293T cells were seeded into a 6-well plate and incubated at 37°C for 24 h. Next, transfection with complexes containing 1,000 ng CHIKV-encoded gene-expressing plasmids, 500 ng IFN-β-Luc vectors, 100 ng β-galactosidase (β-gal)-expressing vectors, and 500 ng each of signaling molecule-expressing plasmid was performed using PEI at a DNA:PEI ratio of 1:2 in a total of 200 μl of Opti-MEM (Thermo Fisher Scientific). At 24 h post-transfection, the transfected cells were lysed with 1× reporter assay lysis buffer (Promega) containing 1× protease inhibitor cocktail (Millipore Sigma). After incubation on ice for 5 min, lysates were harvested and centrifuged at 15,000 ×g and 4°C for 15 min. Then, 25 μl of lysate supernatant and firefly luciferase assay solution were mixed, and the luciferase activity was measured. Similarly, a 25-μl sample and Beta-Glo assay substrate were mixed and incubated at 37°C for 30 min, and then β-gal activity was estimated. Luciferase activity was normalized with the β-gal activity of each sample, which was used to determine the fold induction of the luciferase activity compared to the vector alone control.

### Western Blotting

The lysates were centrifuged at 4°C and 15,000 ×g for 15 min, and the supernatants were harvested for the subsequent assays. The amount of protein in a 10-μl sample was quantified using the Pierce BCA Protein Assay Kit (Thermo Fisher Scientific). Thereafter, 4× Laemmli sample buffer and 2-mercatoethanol were mixed at a 9:1 ratio, and 15 μl of each sample containing the same amount of protein was added. The mixtures were incubated at 100°C for 5 min. Each protein sample was separated using sodium dodecyl sulfate–polyacrylamide gel electrophoresis (SDS-PAGE) and transferred to a nitrocellulose membrane (GE Healthcare Life Science). The transferred membranes were blocked in 5% skim milk (BD, USA) for 1 h and washed three times with 1× TBS-T for 10 min. The membranes were blotted with primary antibodies (1:5,000 mouse monoclonal anti-FLAG, 1:1,000 mouse or rabbit anti-HA, and 1:2,000 anti-GAPDH) at 4°C overnight. Then, the membranes were washed three times with 1× TBS-T and incubated with anti-mouse IgG conjugated with HRP and anti-rabbit IgG conjugated with HRP for 1 h at room temperature. The membranes were treated with either Amersham ECL western blotting detection reagent or Amersham ECL Prime western blotting detection reagent (GE Healthcare Life Science) and exposed on X-ray film (Agfa-Gevaert, Belgium).

### Cell Viability Testing

Transfected cells were treated with EZ-Cytox (DoGenBio, Republic of Korea) with 1/10^th^ of the total culture volume at 24 h post-transfection and incubated at 37°C for 2 h. Then, the optical absorbance of each sample was measured at a wavelength of 450 nm using a SpectraMax iD3 (Molecular Device, USA).

### Statistical Analysis

Data were expressed as the mean ± standard deviation. Representative data of two independent experiments are shown. Statistical significance was analyzed by two-tailed paired Student’s *t*-tests.

## Results

### CHIKV-Encoded Genes Downregulated MDA5 and RIG-I-Mediated Induction of NF-κB Promoter Activities

**Fig. 1:**
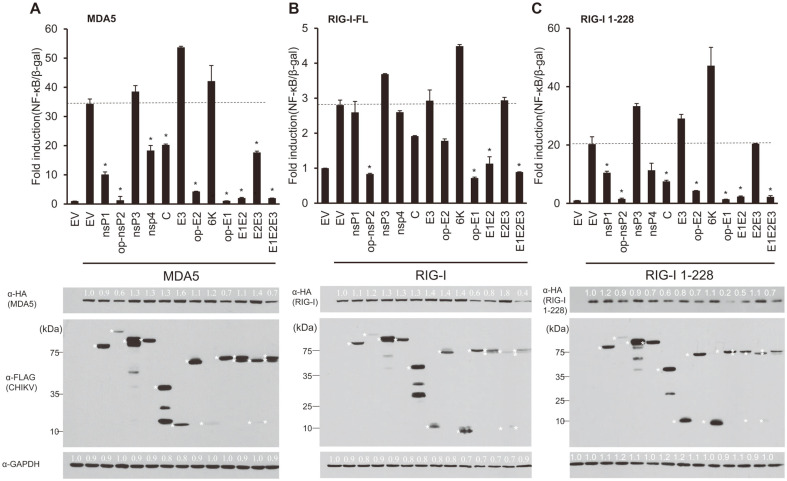
dsRNA-sensing molecules (MDA5 and RIG-I)-mediated induction of NF-κB promoter activities is regulated by Chikungunya virus (CHIKV)-encoded genes. Each individual gene of CHIKV was co-transfected into HEK293T cells with NF-κB-luc, β-gal, and either MDA5 (**A**), RIG-I-FL (**B**), or RIG-I-1-228 (**C**). At 24 h post-transfection, cells were lysed for luciferase activity and western blot, as described in Materials and Methods. Fold induction of the NF-κB promoter over empty vector control is plotted as mean ± standard deviation (upper panels) and its statistical significance was analyzed by two-tailed Student’s t-tests (*p < 0.05). Proteins were resolved by SDS-PAGE and immunoblotted using the indicated antibodies (lower panels). Viral proteins were detected using anti-FLAG antibodies. Asterisks indicate viral genes tagged with N-terminal 3X FLAG. HA-tagged MDA5, RIG-I, and RIG-I-1-228 were detected using anti-HA antibodies. Expression levels of MDA5, RIG-I, and RIG-I-1-228 were normalized to those of GAPDH. Representative data are shown from two independent experiments.

To investigate whether CHIKV-encoded genes regulate MDA5- or RIG-I-mediated induction of NF-κB, all CHIKV proteins were cloned into an expression vector fused with a tag (3X FLAG) and a spacer (3X GGGGS). As the protein expression levels of nsP2, E1, and E2 were initially low to undetectable, it was enhanced by codon optimization (Bionix, Republic of Korea). Many CHIKV-encoded proteins seem to inhibit MDA5- and RIG-I-mediated activation of NF-κB promoter. Among others, both nsP2 and glycoproteins (E1 and E2) strongly downregulated MDA5-induced NF-κB promoter activities by over 80%, presumably by decreasing the protein levels of MDA5 by 40% to 80%, respectively (Fig. 1A). Interestingly, E1, but not nsP2, also decreased the protein levels of RIG-I-FL by 40% while both of them seemed to be able to inhibit RIG-I-mediated induction of NK-κB promoter activities to a similar extent (Figs. 1B and 1C). In addition, nsP1 and E2 glycoproteins also seem to be involved in the RIG-I-like receptors (RLR)-mediated induction of NF-κB promoter activation albeit in a differential manner; MDA5-mediated activation of the NF-κB promoter was decreased by 70% by nsP1 and 85%by E2 according to the luciferase assay whereas RIG-I-FL-induced activation of the NF-κB promoter was inhibited by neither nsP1 or E2.

As E1 and E2 form heterodimers and E3 functions to stabilize them in the host cells, dimeric (E1/E2 or E2/E3) or trimeric (E1/E2/E3) combinations were also screened for their effect(s) on the MDA5- or RIG-I-FL-mediated induction of NF-κB. The E1/E2 heterodimer and the trimer (E1/E2/E3) were able to inhibit the activation of NF-κB by MDA5 and RIG-I-FL over 90% (Fig. 1A). RIG-I-1-228 is a constitutively active form of RIG-I that only expresses the CARD domain of the full-length RIG-I (RIG-I-FL) [19]. RIG-I-1-228-mediated NF-κB activation was suppressed by nsP1 (50%), nsP2 (95%), E2 (80%), and E1 (95%). In addition, RIG-I-1-228 protein levels were greatly reduced by E1 (80%). Therefore, it is conceivable that nsP2 and E1/E2 target the CARD domains of RIG-I (Fig. 1C).

### MAVS-Mediated Induction of the NF-κB Promoter Was Effectively Suppressed by nsP2, E2, and E1

**Fig. 2:**
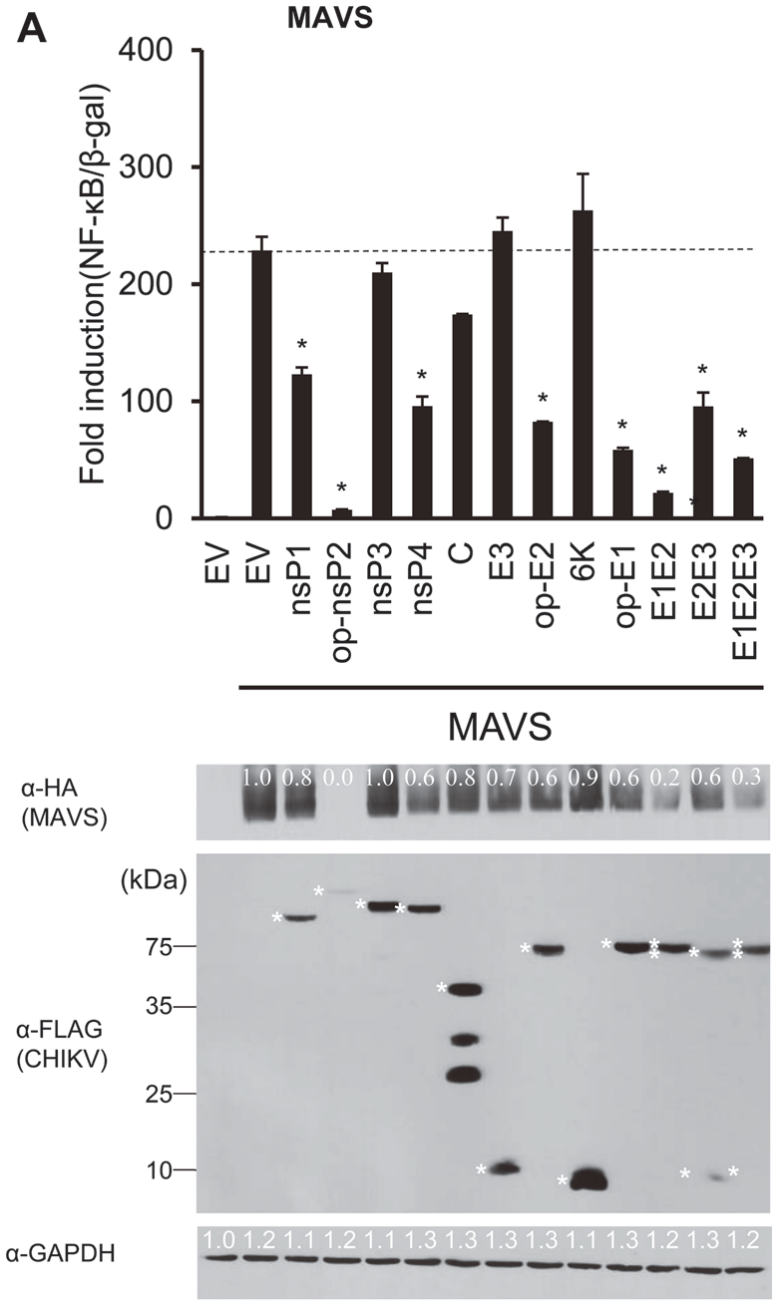
Mitochondrial antiviral-signaling (MAVS), which mediates the activation of nuclear factor kappa B (NF-κB), was modulated by CHIKV-encoded proteins. HEK293T cells were co-transfected with MAVS, each of the CHIKV-encoded genes, NF-κB-luc, and β-gal (control). Fold induction of NF-κB by MAVS in the presence/absence of each individual CHIKV-encoded protein was plotted (upper panel). MAVS (59.2 kDa) was detected using anti-MAVS antibodies (lower panel), and its expression levels were normalized to that of GAPDH (35.8 kDa). Representative data are shown from two independent experiments. Statistical significance was determined by two-tailed Student’s t-tests (*p < 0.05).

To determine whether MAVS-induced NF-κB is suppressed by CHIKV proteins, we transfected each CHIKV-encoded gene, MAVS, NF-κB-luc, and β-gal into HEK293T cells. The results show that MAVS-mediated activation of NF-κB was strongly inhibited by nsP2 (95%), E2 (65%), and E1 (70%) (Fig. 2). In addition, combinations of E1/E2, E2/E3, and E1/E2/E3 significantly inhibited NF-κB promoter activation by MAVS. It is interesting to note that MAVS protein levels were completely inhibited by nsP2 while E1 and E2 were able to significantly downregulate them as well (80% and 40%, respectively).

### TBK1/IKKε-Induced NF-κB Responses Were Inhibited by CHIKV-Encoded Genes

**Fig. 3:**
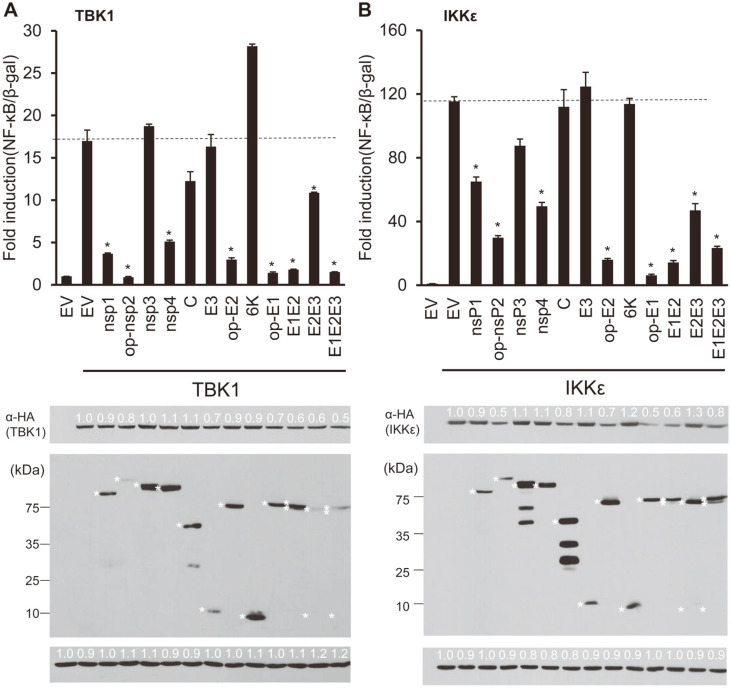
TBK1 and IKKε attenuated NF-κB activation via CHIKV proteins. Following co-transfection with each individual viral gene, NF-κB-Luc, β-gal, and either TBK1 (**A**) or IKKε (**B**), HEK293T cells were incubated in a humidifying incubator for 24 h. Then, the cells were lysed and analyzed by luciferase assay (upper panel) and western blotting (lower panel). HA-tagged TBK1 (86.0 kDa) and IKKε (82.8 kDa) were detected using anti-HA antibodies, and their expression levels were normalized to that of GAPDH (35.8 kDa). Representative data are shown from two independent experiments. Statistical significance was evaluated using two-tailed Student’s t-tests (*p < 0.05).

Activation of TBK1 and IKKε has been shown to promote type I IFN responses. To determine if CHIKV-encoded proteins regulate TBK1/IKKε-mediated activation of IFN-β responses, HEK293T cells were transfected with TBK1- or IKKε-expressing construct, each of the viral genes, NF-κB promoter-expressing plasmid, and β-gal (control). Interestingly, many CHIKV-encoded proteins were able to inhibit TBK1-mediated (nsP2 (95%), E2 (80%), E1 (90%)) and IKKε-mediated activation of the NF-κB promoter (nsP2 (75%), E2 (85 %), E1 (95%))(Fig. 3). Levels of inhibition by CHIKV proteins were correlated with reductions of TBK1/IKKε protein levels, suggesting that CHIKV-encoded proteins seem to downregulate TBK1 and IKKε expression levels by as yet unknown mechanisms.

### MDA5-Induced Activation of the IFN-β and NF-κB Promoter Was Suppressed by nsP2 in a Dose-Dependent Manner

**Fig. 4:**
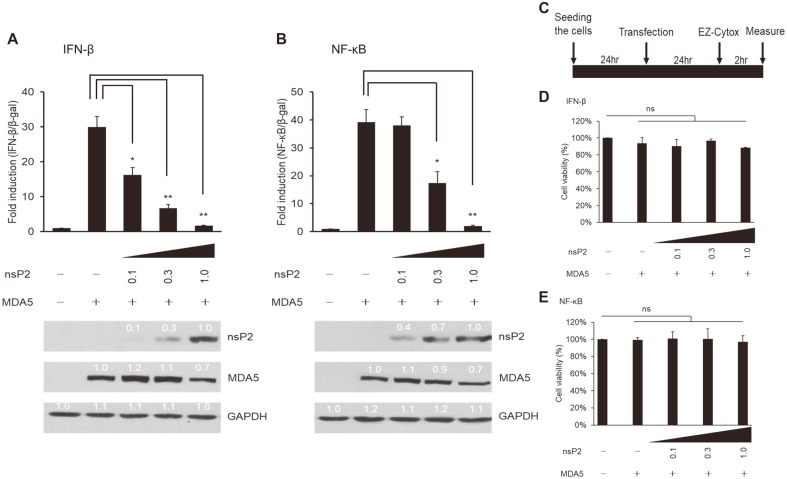
MDA5-mediated induction of interferon (IFN)-β and NF-κB promoter activity was suppressed by CHIKV-nsP2 in a dose-dependent manner. HEK293T cells were co-transfected with CHIKV-nsP2, MDA5, β-gal (control), and either IFN-β-Luc (**A**) or NF-κB-Luc (**B**). Note that nsP2 was transfected at an increasing amount (0.1, 0.3, or 1.0 μg). Luciferase assay and western blotting results are shown in the top and bottom panels, respectively. HA-tagged MDA5 was detected using anti-HA, and nsP2 was detected using anti-FLAG antibodies. Cell viability was assessed using EZ-Cytox (D and E). A schematic description of the experimental method is shown in (**C**). Representative data are shown from two independent experiments. Statistical significance was evaluated using two-tailed Student’s t-tests (*p < 0.05; **p < 0.01).

To examine the kinetics of nsP2-mediated inhibition of the MDA5/RIG-I pathway, HEK293T cells were co-transfected with 0.1, 0.3, or 1.0 μg of nsP2 and MDA5 (Figs. 4A and 4B). Induction of IFN-β and NF-κB promoter activities was determined by luciferase assays. In the presence of an increasing amount of nsP2 proteins, IFN-β and NF-κB promoter activities were inhibited in a dose-responsive manner. More importantly, MDA5 protein levels were downregulated accordingly (Fig. 4), strongly suggesting that nsP2 may directly or indirectly suppress MDA5, leading to reduction of its protein levels, and thus, decrease in the downstream signaling. Ez-Cytox assays demonstrated that overexpression of nsP2 did not adversely affect cell viability (Figs. 4C-4E), suggesting that nsP2-mediated suppression of the signaling pathway may have specific underlying mechanisms that are now being investigated.

### Inhibitory Activity of nsP2 Was Not Affected by the Presence/Absence and Position of a 3X FLAG Tag

**Fig. 5:**
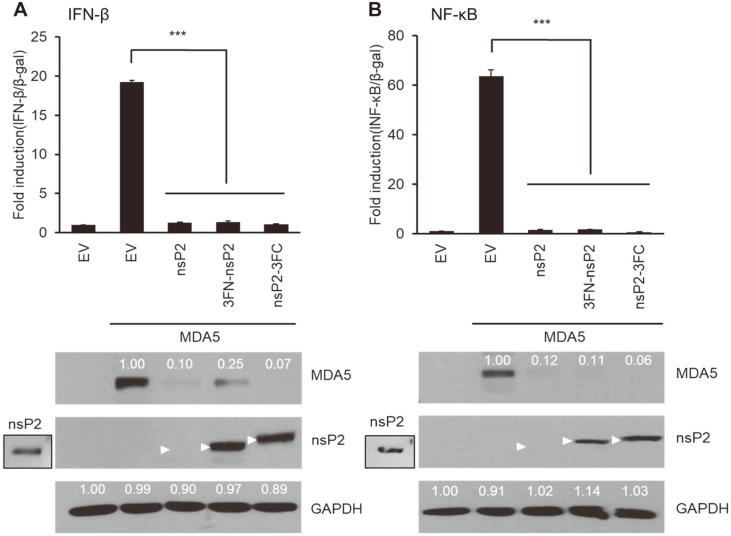
The presence/absence or the position of a tag (3X FLAG) on nsP2 did not affect its antagonism of NF-κB promoter activation. Native nsP2 was used or it was fused to a FLAG either at the N- or C-terminus. Fold induction of MDA5-induced IFN-β (**A**) or NF-κB (**B**) promoter activity in the presence/absence of an expression tag is plotted (upper panels). Protein expression levels were assessed by western blotting (bottom panels). Arrowheads indicate the nsP2 protein. Transfection of the native nsP2 without fusion with an expression tag displayed weak expression, and thus it was over-exposed and shown in the inlet (lower panels). One representative experiment is shown from two independent experiments. Statistical significance was determined by two-tailed Student’s t-tests (*p < 0.05; **p < 0.01; ***p < 0.001).

We used three types of nsP2-expressing constructs: no tag, N-term (3FN), and C-term (3FC) tag (Figs. 5A and 5B). It is known that the presence/absence or position of a tag may have influence on the functions of protein to a varying degree. Our results clearly show that nsP2 was able to inhibit MDA5-mediated activation of NF-κB promoter activities regardless of the position of the 3X FLAG tag (Fig. 5), suggesting that nsP2 is a bona fide antagonist of the MDA5/RIG-I pathway.

## Discussion

Pattern-recognition receptors (PRRs) play a leading role in the innate immune system. PRRs recognize conserved molecular motifs of pathogens such as bacteria and viruses through their distinctive molecular patterns. PRRs are divided into four families: toll-like receptors (TLRs), nucleotide-binding oligomerization domain-like receptors (NLRs), C-type lectin receptors (CLRs), and RLRs [20]. These receptors on the cell surface recognize pathogens, activating innate immune responses. PRR recognition of viral dsRNA by the RLRs (MDA5 and RIG-I) [21] induces the expression of IFNs and pro-inflammatory cytokines via a cascade of signaling events. MDA5 and RIG-I activation upon the recognition of viral dsRNA induces conformational changes of MAVS proteins through CARD–CARD interactions, leading to the interaction with IKKε and TBK1 [22] and then eventually phosphorylation of the transcription factors IRF3 and IRF7. Phosphorylated IRF3 and IRF7 form dimer complexes that are translocated into the nucleus to activate NF-κB, thereby inducing the production of IFNs. The IKK complex is a required component of the canonical NF-κB signaling pathway and consists of two catalytic subunits [23]. TBK1 acts as a downstream kinase mediating dsDNA-mediated IRF3 and NF-κB signaling [24].

The inhibition or downregulation of NF-κB activation through viral proteins has been reported: 1) hepatitis C virus protein is known to diminish NF-KB activation [25], 2) the classical swine fever virus non-structural 5A protein suppresses the poly (I:C)-induced NF-κB signaling pathway [26], 3) the Middle East respiratory syndrome coronavirus-encoded accessory proteins ORF4a and ORF4b or ORF8b have been reported to antagonize NF-κB activation [7], 4) the Myxoma virus M013 protein has been demonstrated to inhibit the NF-κB signaling pathway via direct binding to ASC1 and NF-κB1 [27].

Alphavirus nsP2 has been shown to antagonize antiviral responses including host transcriptional shut-off [28] and downstream type I/II IFN-induced JAK-STAT signaling, resulting in the inhibition of STAT1 phosphorylation and nuclear translocation [29, 30] and ultimately the blockage of type I IFN expression [1]. However, the effects of alphavirus interruption of the canonical NF-κB signaling pathway remain poorly understood. In this study, we screened CHIKV genes for antagonism of NF-κB signaling.

Previously, MDA5 and RIG-I have been shown to inhibit alphavirus replication [31]. Therefore, we hypothesized that CHIKV-nsP2 might be involved in downregulating the activities of those cytosolic receptors. We demonstrated that nsP2 and E1/E2 mediated strong inhibition of MDA5- and RIG-I-induced NF-κB (Figs. 1-3) without cell cytotoxic effects (Fig. 4). CHIKV nsP2 suppressed cytosolic immune receptor-induced NF-κB in a dose-dependent manner (Fig. 4) regardless of the presence/absence of an expression tag (3X FLAG), and its position (whether or not N-term or C-term) or C-term (Fig. 5).

Like nsP2, CHIKV-E1 and -E2 and their combination (E1/E2) were found to interfere with NF-κB induction as well. E1 and E2 are glycoproteins that form heterodimers, mediating membrane fusion between viral envelope and cell plasma membrane to initiate infection [32, 33]. However, alphavirus glycoproteins have not been reported as IFN antagonists. Previously, there were just a handful of studies showing that viral envelop proteins are involved in the antagonism of type I IFN signaling: 1) co-expression of Andes virus nucleocapsid protein with a glycoprotein precursor was found to suppress IFN-β signaling [34], 2) CHIKV E1 and E2 strongly inhibited MDA5/RIG-I-mediated induction of promoter activation of IFN-β [1] 3) Zika virus-encoded envelop protein (E) seems to suppress TBK1-mediated induction of promoter activation of IFN-β [35] as well as NF-κB [36]. Our results showed that CHIKV-encoded E1, E2 and E1/E2 dimer mediate strong antagonism of MDA5/RIG-I-mediated induction of NF-κB promoter activities (Figs. 1-3). Thus, it is tempting to hypothesize that CHIKV-E1 and -E2 proteins add to the list of virus envelop proteins that inhibit type I IFN induction.

**Fig. 6:**
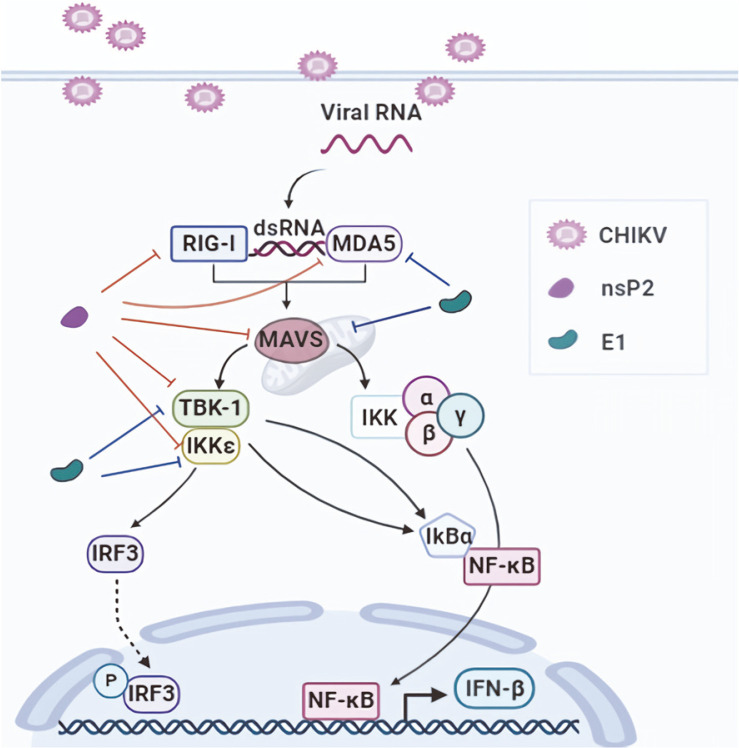
Schematic summary of inhibition of MDA5/RIG-I-mediated NF-κB induction by CHIKV-encoded genes. Signaling pathways for the MDA5/RIG-I-mediated activation of NF-κB are shown. Signaling molecules significantly inhibited by nsP2 and E1 are indicated. CHIKV stands for Chikungunya virus. The figures were prepared using the services of Biorender.com (www.biorender.com)

Taken together, the data presented in the current study provide evidence that nsP2, E1, and E2 exhibit inhibitory activity that may result in the dampening of NF-κB activation (summarized in Fig. 6). Further studies are warranted to elucidate the potential mechanisms of nsP2 and E1/E2-mediated inhibition of type I IFN signaling. Detailed description of the functions of those CHIKV-encoded proteins will provide insight into the development of effective therapeutics and preventive vaccines against CHIKV.

## Figures and Tables

**Table 1:** Primer sequences used in this study.

Primer name	Sequence (5’-3’)
ChiKV-nsp1-F	GGCCTGCAGGATGGATCCTGTGTACGTGGA
ChiKV-nsp1-R	CCGGGCCCTTATGCGCCCGCTCTGTCC
ChiKV-op-nsp2-F	GGCCTGCAGGGGAATAATAGAGACTCCGAGAGG
ChiKV-op-nsp2-R	CCGGGCCCTTAGGTGACCTGTCCTACGAAG
ChiKV-nsp3-F	GGCCTGCAGGCGAGCAGGATGTGCACCGT
ChiKV-nsp3-R	CCGGGCCCTTACCTGTCTAGTCATAACTCGTCGTC
ChiKV-nsp4-F	GGCCTGCAGGGCAGGTGGGTATATATTCTCGTCG
ChiKV-nsp4-R	CCGGGCCCTTATTTAGGACCGCCGTACAAAG
ChiKV-C-F	GGCCTGCAGGATGGAGTTCATCCCAACCCA
ChiKV-C-R	CCGGGCCCTTAACTCCACTCTTCGGCCCC
ChiKV-op-E3-F	GGCCTGCAGGCTTGCCATCCCAGTTATGTGC
ChiKV-op-E3-R	CCGGGCCCTTAACCGCCAGCGACGTAGC
ChiKV-op-E2-F	GGCCTGCAGGACCAAGGACAACTTCAATGTC
ChiKV-op-E2-R	CCGGGCCCTTATTTAGCTGTTCTGATGCAGC
ChiKV-6K-F	GGCCTGCAGGGCGGCCACATACCAAGAGG
ChiKV-6K-R	CCGGGCCCTTAGTACGCGCTCACAGTGTGG
ChiKV-op-E1-F	GGCCTGCAGGGAACACGTAACAGTGATCCCG
ChiKV-op-E1-R	CCGGGCCCTTAGTGCCTGCTGAACGACA

(Dotted underline: restriction enzyme sequences, solid underline: CHIKV sequences. Note that when CHIKV gene sequences were codon-optimized for *Homo sapiens*, gene names are prefixed with “op-“).

## References

[1] Bae S, Lee JY, Myoung J (2019). Chikungunya virus-encoded nsP2, E2 and E1 strongly antagonize the interferon-beta signaling pathway. J. Microbiol. Biotechnol..

[2] Burt FJ, Chen W, Miner JJ, Lenschow DJ, Merits A, Schnettler E (2017). Chikungunya virus: an update on the biology and pathogenesis of this emerging pathogen. Lancet Infect. Dis..

[3] Bustos Carrillo F, Collado D, Sanchez N, Ojeda S, Lopez Mercado B, Burger-Calderon R (2019). Epidemiological evidence for lineage-specific differences in the risk of inapparent chikungunya virus infection. J. Virol..

[4] Zhang YN, Deng CL, Li JQ, Li N, Zhang QY, Ye HQ (2019). Infectious chikungunya virus (CHIKV) with a complete capsid deletion: a new approach for a CHIKV vaccine. J. Virol..

[5] Kato H, Takeuchi O, Sato S, Yoneyama M, Yamamoto M, Matsui K (2006). Differential roles of MDA5 and RIG-I helicases in the recognition of RNA viruses. Nature.

[6] Lee JY, Bae S, Myoung J (2019). Middle East respiratory syndrome coronavirus-encoded ORF8b strongly antagonizes IFN-beta promoter activation: its implication for vaccine design. J. Microbiol..

[7] Lee JY, Bae S, Myoung J (2019). Middle east respiratory syndrome coronavirus-encoded accessory proteins impair MDA5-and TBK1-mediated activation of NF-kappaB. J. Microbiol. Biotechnol..

[8] Zeng W, Sun L, Jiang X, Chen X, Hou F, Adhikari A (2010). Reconstitution of the RIG-I pathway reveals a signaling role of unanchored polyubiquitin chains in innate immunity. Cell.

[9] Lee JY, Kim SJ, Myoung J (2019). Middle east respiratory syndrome coronavirus-encoded ORF8b inhibits RIG-I-like receptors in a differential mechanism. J. Microbiol. Biotechnol..

[10] Myoung J, Lee JY, Min KS (2019). Methyltransferase of a cell culture-adapted hepatitis E inhibits the MDA5 receptor signaling pathway. J. Microbiol..

[11] Myoung J, Lee SA, Lee HR (2019). Beyond viral interferon regulatory factors: Immune evasion strategies. J. Microbiol. Biotechnol..

[12] Ramos HJ, Gale M (2011). RIG-I like receptors and their signaling crosstalk in the regulation of antiviral immunity. Curr. Opin. Virol..

[13] Myoung J, Min K (2019). Dose-dependent inhibition of melanoma differentiation-associated gene 5-mediated activation of type I interferon responses by methyltransferase of hepatitis E virus. J. Microbiol. Biotechnol..

[14] Park BJ, Jung ST, Choi CS, Myoung J, Ahn HS, Han SH (2018). Pathogenesis of human norovirus genogroup II genotype 4 in post-weaning gnotobiotic pigs. J. Microbiol. Biotechnol..

[15] Kang S, Choi C, Choi I, Han KN, Rho SW, Choi J (2018). Hepatitis E virus methyltransferase inhibits type I interferon induction by targeting RIG-I. J. Microbiol. Biotechnol..

[16] Kim E, Myoung J (2018). Hepatitis E virus papain-like cysteine protease inhibits type I interferon induction by down-regulating melanoma differentiation-associated gene 5. J. Microbiol. Biotechnol..

[17] Ahn DG, Shin HJ, Kim MH, Lee S, Kim HS, Myoung J (2020). Current status of epidemiology, diagnosis, therapeutics, and vaccines for novel coronavirus disease 2019 (COVID-19). J. Microbiol. Biotechnol..

[18] Lee J, Bae S, Myoung J (2019). Generation of full-length infectious cDNA clones of middle east respiratory syndrome coronavirus. J. Microbiol. Biotechnol..

[19] Saito T, Hirai R, Loo YM, Owen D, Johnson CL, Sinha SC (2007). Regulation of innate antiviral defenses through a shared repressor domain in RIG-I and LGP2. Proc. Natl. Acad. Sci. USA.

[20] Areschoug T, Gordon S (2008). Pattern recognition receptors and their role in innate immunity: focus on microbial protein ligands. Contrib. Microbiol..

[21] Brisse M, Ly H (2019). Comparative Structure and Function Analysis of the RIG-I-Like Receptors: RIG-I and MDA5. Front. Immunol..

[22] Seth RB, Sun L, Ea CK, Chen ZJ (2005). Identification and characterization of MAVS, a mitochondrial antiviral signaling protein that activates NF-kappaB and IRF 3. Cell.

[23] Hinz M, Scheidereit C (2014). The IkappaB kinase complex in NF-kappaB regulation and beyond. EMBO Rep..

[24] Abe T, Barber GN (2014). Cytosolic-DNA-mediated, STING-dependent proinflammatory gene induction necessitates canonical NF-kappaB activation through TBK1. J. Virol..

[25] Zhang L, Alter HJ, Wang H, Jia S, Wang E, Marincola FM (2013). The modulation of hepatitis C virus 1a replication by PKR is dependent on NF-κB mediated interferon beta response in Huh7.5. cells. Virology.

[26] Dong XY, Tang SQ (2016). Classical swine fever virus NS5A protein changed inflammatory cytokine secretion in porcine alveolar macrophages by inhibiting the NF-kappaB signaling pathway. Virol. J..

[27] Garg RR, Jackson CB, Rahman MM, Khan AR, Lewin AS, McFadden G (2019). Myxoma virus M013 protein antagonizes NF-kappaB and inflammasome pathways via distinct structural motifs. J. Biol. Chem..

[28] Akhrymuk I, Kulemzin SV, Frolova EI (2012). Evasion of the innate immune response: the Old World alphavirus nsP2 protein induces rapid degradation of Rpb1, a catalytic subunit of RNA polymerase II. J. Virol..

[29] Fros JJ, Liu WJ, Prow NA, Geertsema C, Ligtenberg M, Vanlandingham DL (2010). Chikungunya virus nonstructural protein 2 inhibits type I/II interferon-stimulated JAK-STAT signaling. J. Virol..

[30] Breakwell L, Dosenovic P, Karlsson Hedestam GB, D'Amato M, Liljestrom P, Fazakerley J (2007). Semliki Forest virus nonstructural protein 2 is involved in suppression of the type I interferon response. J. Virol..

[31] Akhrymuk I, Frolov I, Frolova EI (2016). Both RIG-I and MDA5 detect alphavirus replication in concentration-dependent mode. Virology.

[32] Strauss JH, Strauss EG (1994). The alphaviruses: gene expression, replication, and evolution. Microbiol. Rev..

[33] Kuo SC, Chen YJ, Wang YM, Tsui PY, Kuo MD, Wu TY (2012). Cell-based analysis of Chikungunya virus E1 protein in membrane fusion. J. Biomed. Sci..

[34] Levine JR, Prescott J, Brown KS, Best SM, Ebihara H, Feldmann H (2010). Antagonism of type I interferon responses by new world hantaviruses. J. Virol..

[35] Ngueyen TTN, Kim SJ, Lee JY, Myoung J (2019). Zika Virus proteins NS2A and NS4A Are major antagonists that reduce IFN-beta promoter activity induced by the MDA5/RIG-I signaling pathway. J. Microbiol. Biotechnol..

[36] Lee JY, Nguyen TTN, Myoung J (2020). Zika Virus-encoded NS2A and NS4A strongly downregulate NF-kappaB promoter activity. J. Microbiol. Biotechnol..

